# Endoscopic surgical treatment of sinonasal Schneiderian papillomas: our experience

**DOI:** 10.1007/s00405-026-10420-8

**Published:** 2026-07-19

**Authors:** Salvatore Poma, Andrea Comparetto, Domiziana Picone, Antonino Milia, Valerio Patti, Mauro Sergi, Cuffaro Luana, Domenico Michele Modica, Alberto Fucarino, Alessandro Pitruzzella

**Affiliations:** 1 Department of Otorhinolaryngology, Villa Sofia-Cervello Hospital, Palermo, 90146 Italy; 2 Department of Biomedicine, Neurosciences and Advanced Diagnostics (BiND), University of Palermo, Palermo, 90127 Italy; 3 General surgeon, freelance, Palermo, 90146 Italy; 4 Department of Theoretical and Applied Sciences, eCampus University, Novedrate, 22060 Italy

**Keywords:** Schneiderian papilloma, Inverted papilloma, Sinonasal tumors, Endoscopic sinus surgery, Recurrence, Metachronous carcinoma

## Abstract

**Background:**

Sinonasal Schneiderian papillomas are uncommon epithelial tumors. Although benign, they may behave aggressively, recur after incomplete resection, and, particularly in the inverted subtype, be associated with carcinoma. Endoscopic surgery is now widely used when the site of attachment can be adequately exposed and treated.

**Objective:**

To review recurrence, morbidity, attachment-site documentation, and metachronous carcinoma in a single-surgeon endoscopic series of sinonasal Schneiderian papillomas treated with an attachment-site-oriented technique.

**Methods:**

We retrospectively reviewed 71 patients with histologically confirmed Schneiderian papilloma treated exclusively by endoscopic surgery between January 2017 and December 2023. Preoperative work-up included nasal endoscopy, CT, and MRI. Disease extent was classified according to the Krouse system. Surgery was planned according to tumor extension and the suspected attachment site. Follow-up was based on serial endoscopic examinations; annual MRI was advised for long-term surveillance. Median follow-up was 46 months.

**Results:**

Krouse staging identified 3 T1, 24 T2, 40 T3, and 4 T4 lesions. Histology included 58 inverted, 8 exophytic, and 5 oncocytic papillomas. Recurrence occurred in 3 patients (4.2%), all with inverted papilloma and advanced-stage disease (T3–T4). Complications were observed in 4 patients (5.6%), including three arterial bleeding episodes and one cerebrospinal fluid leak. One patient developed metachronous squamous cell carcinoma during follow-up.

**Conclusion:**

In this single-surgeon series, attachment-site-oriented endoscopic surgery was followed by few recurrences and acceptable morbidity during the available follow-up. Recurrences were observed only in advanced-stage inverted papillomas. These findings support careful treatment of the attachment site and long-term surveillance, while remaining limited by the retrospective design and heterogeneous follow-up.

## Introduction

Sinonasal Schneiderian papillomas arise from the Schneiderian membrane and are classically divided into inverted, exophytic, and oncocytic subtypes [1–3]. Although benign, they may produce bone remodeling, recur after incomplete excision, and show dysplasia or carcinoma, particularly in the inverted subtype [3, 4].

Surgical treatment has progressively shifted from external approaches to endoscopic techniques. In contemporary endoscopic surgery, the key step is the identification and treatment of the attachment site: the tumor is debulked to expose the pedicle, the involved mucosa is removed subperiosteally, and the underlying bone is drilled or cauterized when appropriate [5–7].

Recurrence remains an important problem, particularly in extensive inverted papilloma. Malignancy may be synchronous or may appear later during surveillance, so follow-up should not be limited to the early postoperative period [8–11]. In clinical follow-up, stage, histologic subtype, and attachment site are therefore relevant for risk assessment.

This study reviews a single-surgeon endoscopic series of sinonasal Schneiderian papillomas managed with an attachment-site-oriented approach. We focused on recurrence patterns, morbidity, metachronous carcinoma, and the completeness of attachment-site documentation in a real-world retrospective cohort.

## Materials and methods

### Study design and population

This retrospective observational study was reported in accordance with the STROBE recommendations for cohort studies [12]. The cohort included 71 consecutive patients with histologically confirmed sinonasal Schneiderian papilloma who underwent exclusively endoscopic surgical treatment between January 2017 and December 2023 at a tertiary otolaryngology department.

Patients were eligible when the diagnosis of Schneiderian papilloma was confirmed histologically and the lesion was resected endoscopically at the study institution. Patients with incomplete clinical records or no postoperative follow-up were excluded.

The cohort comprised 43 males and 28 females, with a mean age of 60.2 years (range, 27–93 years). At presentation, 68 patients (95.8%) had primary disease, whereas 3 patients (4.2%) had previously undergone surgery elsewhere and were referred with recurrent disease.

The study was approved by the local ethics committee as a retrospective observational study. Full ethics details are reported on the separate title page. All data were anonymized before analysis.

### Preoperative assessment

All patients underwent nasal endoscopy, CT, and MRI before surgery. Imaging was used to define tumor extension, evaluate bone changes, and identify the likely site of attachment.

Lesions were staged according to the Krouse classification [13]. The same staging system was used for all Schneiderian papilloma subtypes to provide a uniform description of disease extent. Staging was taken from the contemporaneous clinical records. In the T4 subgroup, the available retrospective data confirmed the recorded stage but did not consistently preserve the individual criteria used for each case; this limitation was considered when interpreting stage-related findings.

### Surgical technique

All operations were performed with an attachment-site-oriented endoscopic technique. After debulking the exophytic portion of the tumor, the attachment was sought using both the preoperative imaging and the intraoperative endoscopic findings.

The mucosa at the attachment site was removed subperiosteally. When the bone appeared involved or thickened, it was drilled or cauterized. Completeness of surgery was judged by endoscopic exposure and treatment of the attachment site; frozen-section margin assessment was not used routinely.

The extent of surgery was adapted to the location and spread of disease. Procedures included standard endoscopic sinus surgery, endoscopic partial maxillectomy, Draf procedures, and combined approaches when required. Because the operative records used FESS and ESS terminology inconsistently, these were grouped as standard endoscopic sinus surgery.

### Follow-up and outcome measures

Patients were examined endoscopically at 1 and 3 months after surgery. During the first year, follow-up visits were scheduled every 6 months and then annually. Annual MRI was recommended for long-term surveillance, although imaging follow-up was not available in all patients.

Recurrence was defined as endoscopic and/or radiological evidence of tumor recurrence after apparently complete removal. Complications were recorded when documented in the operative or postoperative clinical notes.

Median follow-up was 46 months (range, 12–92 months), and 24 patients (33.8%) had at least 5 years of surveillance.

### Statistical analysis

Continuous variables are presented descriptively. Categorical variables are reported as numbers and percentages. Because only three recurrences occurred, no comparative or multivariable analysis was attempted.

## Results

Seventy-one patients with sinonasal Schneiderian papilloma underwent endoscopic surgery during the study period. There were 43 males (60.6%) and 28 females (39.4%), with a mean age of 60.2 years (range, 27–93 years). Sixty-eight patients (95.8%) had primary disease, and 3 patients (4.2%) had previously been operated on elsewhere and were referred with recurrent disease.

Krouse staging showed 3 T1 lesions (4.2%), 24 T2 lesions (33.8%), 40 T3 lesions (56.3%), and 4 T4 lesions (5.6%). Final histology showed 58 inverted papillomas (81.7%), 8 exophytic papillomas (11.3%), and 5 oncocytic papillomas (7.0%). The three patients referred with recurrent disease were included in the cohort because they underwent the index endoscopic procedure at our institution; postoperative recurrence was counted only when disease reappeared after that procedure. Baseline characteristics and outcomes are shown in Table 1.

**Table 1:** Baseline characteristics, outcomes, and documented attachment sites of the study cohort (*n* = 71)

Variable	Value
Number of patients	71
Sex (male/female)	43 (60.6%) / 28 (39.4%)
Mean age (range)	60.2 years (27–93)
Primary cases	68 (95.8%)
Recurrent cases at presentation	3 (4.2%)
Median follow-up (range)	46 months (12–92)
Follow-up ≥ 5 years	24 (33.8%)
Krouse stage T1	3 (4.2%)
Krouse stage T2	24 (33.8%)
Krouse stage T3	40 (56.3%)
Krouse stage T4	4 (5.6%)
Inverted papilloma	58 (81.7%)
Exophytic papilloma	8 (11.3%)
Oncocytic papilloma	5 (7.0%)
Recurrence	3 (4.2%)
Complications	4 (5.6%)
Metachronous squamous cell carcinoma	1 (1.4%)
Attachment site documented	67 (94.4%)
Uncinate process	11 (16.4%)
Lateral wall of the maxillary sinus	11 (16.4%)
Anterior maxillary wall	8 (11.9%)
Middle turbinate	7 (10.4%)
Other documented attachment sites	30 (44.8%)
Attachment site not available in operative documentation	4 (5.6%)

Percentages for individual attachment-site categories were calculated among cases with documented attachment-site data (*n* = 67). Percentages for all other variables, including unavailable attachment-site documentation, were calculated using the total cohort (*n* = 71)

Table 2 shows histologic subtype and recurrence. Three patients developed postoperative recurrence, giving an overall recurrence rate of 4.2%. All recurrences occurred in inverted papillomas, corresponding to a subtype-specific recurrence rate of 5.1% (3/58). No recurrence was observed in the exophytic or oncocytic subgroups during the available follow-up.

**Table 2:** Histologic subtype distribution and recurrence

Histologic subtype	No. of patients	%	Recurrences	Recurrence rate
Inverted papilloma	58	81.7	3	5.1%
Exophytic papilloma	8	11.3	0	0%
Oncocytic papilloma	5	7.0	0	0%
Total	71	100	3	4.2%

All three recurrences occurred in advanced-stage disease (T3–T4) and appeared between 1 and 3 years after surgery. Each patient underwent revision endoscopic surgery, with no persistent disease documented in the available follow-up. The recurrence details that could be extracted reliably from the retrospective records are summarized in Table 3.

**Table 3:** Summary of postoperative recurrences after the index endoscopic procedure

Variable	Available data
Number of recurrences	3/71 (4.2%)
Histologic subtype	All inverted papilloma
Krouse stage category	All advanced-stage disease (T3–T4)
Time to recurrence	Within 1–3 years after the index procedure
Management	Revision endoscopic surgery in all cases
Outcome after revision	No persistent disease documented in the available follow-up
Case-level attachment site	Not consistently available in the retrospective operative summary

Table 4 reports the procedures recorded in the operative summaries. Detailed procedure subtyping was available for 69 of 71 patients. In the remaining two cases, the records confirmed endoscopic management but did not allow reliable assignment to a specific procedure subtype; these patients were kept in the overall cohort.

**Table 4:** Surgical procedures available in the operative summary

Procedure	*n* (%)
Endoscopic partial maxillectomy type 1	10 (14.5%)
Endoscopic partial maxillectomy type 1b	1 (1.4%)
Endoscopic partial maxillectomy type 2	14 (20.3%)
Endoscopic partial maxillectomy type 2b	5 (7.2%)
Endoscopic partial maxillectomy type 3	8 (11.6%)
Draf I combined with endoscopic partial maxillectomy type 1	1 (1.4%)
Draf IIa	2 (2.9%)
Draf IIb	3 (4.3%)
Draf III	2 (2.9%)
Standard endoscopic sinus surgery	23 (33.3%)

Detailed procedure subtyping was available for 69/71 patients. Percentages are calculated among patients with documented procedure subtyping (*n* = 69). In two cases, the available records confirmed endoscopic management but did not allow reproducible assignment to a specific procedure subtype

Four patients (5.6%) had complications: three arterial bleeding episodes and one cerebrospinal fluid leak. One patient with T4 maxillary disease developed squamous cell carcinoma during postoperative surveillance.

Attachment sites were explicitly recorded in 67 cases and are reported in Table 1. The most frequent documented sites were the uncinate process and the lateral wall of the maxillary sinus (11/67 cases each, 16.4%), followed by the anterior maxillary wall (8/67, 11.9%) and the middle turbinate (7/67, 10.4%). Attachment site was not available in 4 cases.

## Discussion

This single-surgeon series describes attachment-site-oriented endoscopic management of sinonasal Schneiderian papillomas in routine clinical practice. The overall recurrence rate was 4.2% for the whole cohort and 5.1% among inverted papillomas. These figures are in the lower range of published endoscopic series, but they should be interpreted in the context of the retrospective design, heterogeneous follow-up, and the limited number of recurrence events.

The recurrence rate observed here is comparable with mature endoscopic series that emphasize complete exposure and treatment of the attachment site. Lombardi et al. reported a 5.7% recurrence rate and 9.4% complications in 212 patients treated mainly by endoscopic surgery, with a mean follow-up of 53.8 months [14]. In contrast, Yu et al. reported recurrence in 15.3% of 85 endoscopically treated inverted papillomas, with a median time to recurrence of 22 months; importantly, all recurrent tumors arose at the primary attachment site [15]. In the present cohort, all recurrences also appeared within 1–3 years and were managed endoscopically, supporting the clinical relevance of careful pedicle control rather than suggesting that recurrence risk has been eliminated.

Recurrence was confined to inverted papilloma and to advanced-stage disease (T3-T4). This pattern is consistent with the clinical behavior of inverted papilloma, but the small number of events does not allow a formal risk-factor analysis. No recurrence was seen in exophytic or oncocytic papillomas. These subgroups were small, and the absence of recurrence should not be taken as evidence that surveillance can be relaxed.

From a surgical standpoint, the attachment site remains the practical target of treatment. In most cases in this series, the attachment site was documented and treated by subperiosteal mucosal removal with drilling or cauterization of the underlying bone when required. This is consistent with reports focused on maxillary sinus disease, where recurrence has been linked to difficult-to-access attachment sites and to the adequacy of surgical exposure. Kim et al. reported an overall recurrence rate of 5.8% in 155 maxillary sinus inverted papilloma cases and found the highest recurrence rate for lateral wall origin (15.0%) [16]. The relatively frequent maxillary attachment sites in the present cohort therefore support the need for extended endoscopic access when standard antrostomy is not sufficient.

One patient developed squamous cell carcinoma during follow-up. Because continuity with the preceding papilloma could not be demonstrated, this event is best described as a metachronous carcinoma detected during surveillance, not as proven malignant transformation. Nevertheless, its occurrence is clinically relevant and supports prolonged follow-up, especially in advanced inverted papilloma and in cases where postoperative visualization is limited.

A strength of the study is that all operations were performed by one surgeon using the same endoscopic principles. This reduces variability in surgical decision-making and makes the description of surgical outcomes more coherent. At the same time, it limits external generalizability, because the results depend on experience with extended endoscopic approaches and on the ability to expose and treat the tumor attachment site.

### Limitations

This study has several limitations. Its retrospective design may have introduced selection bias and limited the completeness of data collection. The small number of recurrence events precluded comparative or multivariable analysis, and the findings should therefore be read as descriptive rather than predictive. Follow-up duration was heterogeneous; because only one third of patients had at least 5 years of surveillance, late recurrences may have been underestimated. Imaging surveillance was recommended but was not uniformly available for all patients, which may have reduced detection of asymptomatic recurrence. Histologic margin assessment was not routinely performed, and data on HPV status, smoking history, dysplasia, and detailed margin status were not uniformly available. Detailed postoperative management of complications was not consistently categorized. Finally, procedure subtyping was not reproducibly available for two patients, although all patients were confirmed to have undergone endoscopic treatment.

## Conclusions

In this single-surgeon retrospective series, attachment-site-oriented endoscopic treatment of sinonasal Schneiderian papillomas was followed by few recurrences and acceptable morbidity during the available follow-up, when adequate exposure of the attachment site could be achieved. Recurrence was observed only in advanced-stage inverted papillomas.

The results support careful endoscopic treatment of the attachment site and long-term follow-up, while acknowledging that late recurrence may be underestimated in retrospective series with heterogeneous surveillance.
